# Chromatic acclimation shapes phytoplankton biogeography

**DOI:** 10.1126/sciadv.adr9609

**Published:** 2025-02-19

**Authors:** Francesco Mattei, Anna E. Hickman, Julia Uitz, Louison Dufour, Vincenzo Vellucci, Laurence Garczarek, Frédéric Partensky, Stephanie Dutkiewicz

**Affiliations:** ^1^ Sorbonne Université, CNRS, Laboratoire d’Océanographie de Villefranche, LOV, Villefranche-sur-Mer F-06230, France.; ^2^ School of Ocean and Earth Science, University of Southampton, Southampton, SO14 3ZH, UK.; ^3^ Sorbonne Université, CNRS, Adaptation and Diversity in Marine Environments, Roscoff F-29680, France.; ^4^ Sorbonne Université, CNRS, Institut de la Mer de Villefranche, IMEV, Villefranche-sur-Mer F-06230, France.; ^5^ Department of Earth Atmosphere and Planetary Sciences, Massachusetts Institute of Technology, Cambridge, MA, USA.; ^6^ Center for Sustainability Science and Strategy, Massachusetts Institute of Technology, Cambridge, MA, USA.

## Abstract

Marine photoautotrophs have evolved to exploit the ocean’s variable light conditions, with chromatic acclimators being able to adjust their pigment content to better match the ambient light color. The impact of chromatic acclimation on phytoplankton distribution and competition is not well understood despite its global importance. This study explores chromatic acclimation’s role in shaping the biogeography of *Synechococcus*, a widespread cyanobacterium. We integrated three pigment types into a global ecosystem model: a green-light specialist, a blue-light specialist, and a chromatic acclimator. Laboratory studies defined each type’s specific absorption properties. Our results indicate that chromatic acclimation offers an evolutionary advantage by enabling *Synechococcus* to adapt to varying light environments. This ability to mimic blue- and green-light specialists and enhance absorption at intermediate states, particularly in areas with high seasonal light variations, increases *Synechococcus* distribution and biomass. Thus, chromatic acclimation affects ecosystem functioning and biogeochemical processes in the ocean.

## Teaser

Chromatic acclimation is an advantageous evolutionary trait and influences phytoplankton competition and global distribution.

## INTRODUCTION

Photoautotrophs exploit pigments to harvest light energy and protect themselves from its potential damaging effects. The large spatiotemporal variability of the light resource in aquatic ecosystems compared to terrestrial environments led phytoplankton to evolve unique traits and, notably, an extensive pigment diversity, which enables light absorption from distinct parts of the light spectrum (*1*–*4*). The combination of pigments within a phytoplankton species results from adaptation processes involving long-term evolutionary changes. In addition, photoautotrophs can also undertake rapid and reversible physiological adjustments in the amount of pigments through acclimation processes. A remarkable form of the latter is chromatic acclimation, which confers certain phytoplankton species the ability to reversibly modify the relative concentrations of photosynthetic pigments to better match the ambient light spectral quality, optimizing light absorption and thereby enhancing growth (*5*, *6*). This ability differs from photoacclimation, a process found in all photoautotrophs, which primarily consists of adjusting photosynthetic and/or photoprotective pigment ratios to deal with variations in light quantity (e.g., with depth) (*7*). Chromatic acclimation has evolved multiple times in prokaryotes and eukaryotes, and its occurrence has been documented in freshwater, marine, and terrestrial ecosystems (*6*, *8*–*10*). However, the effect of chromatic acclimation on species distribution remains unclear (*11*, *12*). Here, we illustrate the role of chromatic acclimation in defining marine phytoplankton biogeography. We focus on the cyanobacterium *Synechococcus* since chromatic acclimation has recently been shown to have widespread importance within this genus (*13*).

*Synechococcus* is the second most abundant photosynthetic organism in the ocean, and its distribution encompasses all oceanic biomes from equatorial to subpolar regions (*14*–*16*). This cyanobacterium displays the largest pigment diversity known in a single phytoplankton lineage (*17*, *18*), and its light-harvesting antennae, called phycobilisomes, are composed of rods radiating around a central core (*19*). The rods can bind different chromophorylated proteins with distinct light absorption properties (*20*–*23*), and their multiple possible arrangements generate *Synechococcus* strains with distinct pigmentation hereafter called “pigment types” (PTs). We focused on PTs harboring both the green light–absorbing phycoerythrobilin (PEB) and blue light–absorbing phycourobilin (PUB) chromophores, a combination shared by most marine *Synechococcus* (*13*). Some are specialized in absorbing green light [green specialist (GS)] or blue light [blue specialist (BS)], while others can perform chromatic acclimation [chromatic acclimator (CA)]. The phycobilisomes of the GSs are PEB rich, while the BSs are PUB rich. The CA can reversibly modify its PUB/PEB ratio to better match the ambient light color. When the light field is bluer, CA phycobilisomes are enriched in PUB, whereas a greener light field results in PEB-rich phycobilisomes.

**Fig. 1.**
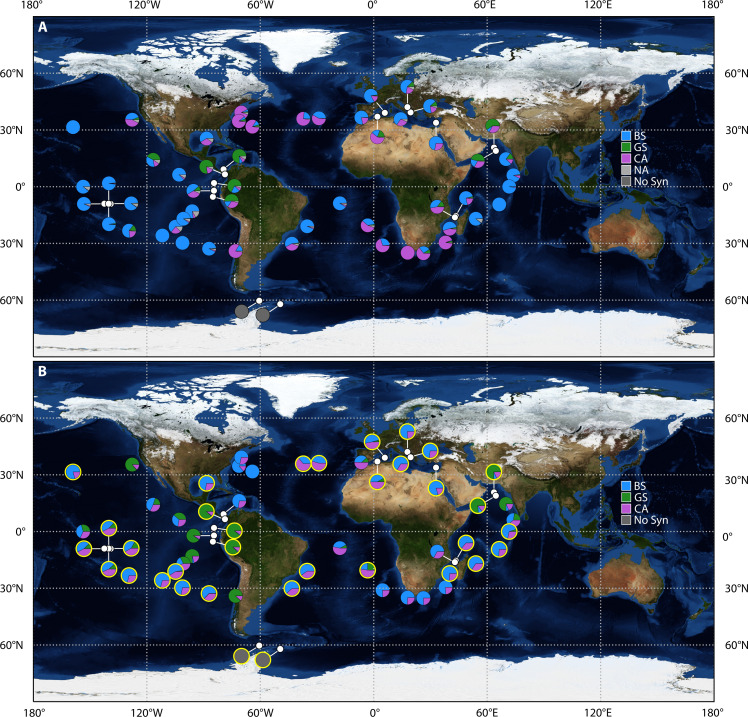
Surface global distribution of *Synechococcus* PTs. (**A**) Relative gene abundance of *Synechococcus* PTs in surface waters estimated on the basis of metagenomic analysis of *Tara* Oceans samples (*13*) and grouped as described elsewhere (*42*). The “NA” label refers to the *Synechococcus* percentage that could not be assigned to any specific PT by the metagenomic analysis, while the “No Syn” label indicates the absence of *Synechococcus* in the *Tara* Oceans data and model simulation. (**B**) Relative abundance of *Synechococcus* PTs in surface waters computed from Darwin simulation biomass for the month at which the samples were collected at each location along the *Tara* Oceans transect. The pie charts circled in yellow represent consistent results in terms of dominant PT between the metagenomic analysis and the modeled biomass. Note that only qualitative comparison is possible because *Tara* Oceans data represent gene abundance, while the model provides biomass. Only the *Tara* Oceans stations within the model domain are displayed (given the coarse resolution, some coastal stations were not resolved in the model). The white dots in both panels depict the coordinates of the *Tara* stations when the pie charts needed to be repositioned to prevent overlap.

The *Tara* Oceans expedition (*24*) revealed distinct biogeographical patterns among *Synechococcus* PTs and provided evidence of CA widespread distribution and large relative abundance (*13*) (Fig. 1A). Here, we investigate the mechanisms by which chromatic acclimation acts on the distributions of the different PTs, including whether a competitive advantage stems from being able to reversibly mimic the specialists’ absorption characteristics or from being able to optimize light harvesting at intermediate acclimation states, potentially enabling a more efficient use of underwater spectral light niches. In addition, we aimed to improve our understanding of *Synechococcus* distribution, crucial for predicting the impact of climate change on these widespread organisms (*15*, *25*–*28*).

We customized a global-scale ecosystem model (Darwin) (*29*–*31*) to explicitly incorporate *Synechococcus* BS, GS, and CA PTs. These types were added to the multiple plankton functional groups and size classes already resolved by the model, which is embedded within a three-dimensional (3D) global biogeochemistry and physics framework. Crucially, the model includes a spectrally resolved light field and each phytoplankton type has specific light absorption characteristics (*29*).

## RESULTS

### Parameterization of *Synechococcus* PTs

**Fig. 2.**
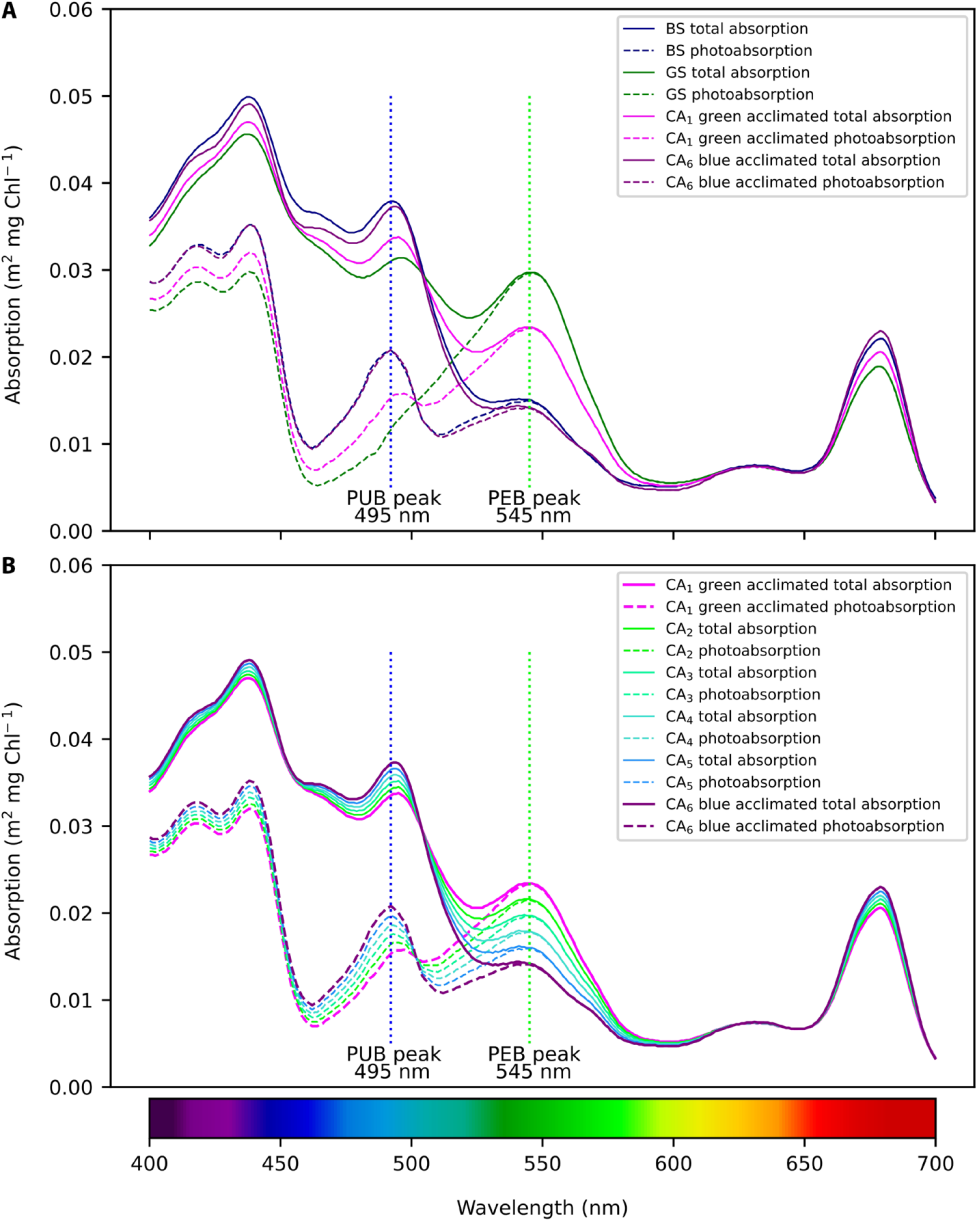
Total and photosynthetic chlorophyll *a*–specific absorption spectra of simulated *Synechococcus* PTs. (**A**) Total (solid lines) and photosynthetic absorption (dashed lines) spectra of GSs (green lines), BSs (blue lines), and CAs acclimated to blue and green light (indicated by purple and pink lines, respectively). (**B**) Total (solid) and photosynthetic (dashed) absorption spectra of the CA acclimated to blue and green light (purple and pink lines, respectively) and intermediate acclimation states. The blue and green vertical dotted lines depict the absorption peak of PUB (495 nm) and PEB (545 nm), respectively. “Total absorption” refers to the absorption by all pigments, and “photosynthetic absorption” refers to absorption by only pigments used for photosynthesis (see Materials and Methods and the Supplementary Materials for details).

The three *Synechococcus* PTs implemented in the Darwin model (BS, GS, and CA) are identical in terms of parameters (such as maximum growth rate and nutrient affinity) and have similar photoacclimation dynamics, but each is characterized by a distinct light absorption spectrum representing the different pigment contents. The CA is allowed to shift its spectrum to optimize the absorption of available blue and green light. We gathered *Synechococcus* PT absorption spectra from the literature (*32*–*34*) and complemented them with additional data from cultures representative of the three PTs to assess the intra- and inter-PT variability. This enabled us to compute the characteristic absorption spectra for the model analogs of the BS, GS, and CA (see Materials and Methods, Supplemental section 1.1, and Fig. 2A). For the CA, we assessed absorption spectra of CA strains measured at different times during the shift from blue to green acclimation (*35*) (Supplemental section 1.2). The different CA acclimation states were simulated in the model as six distinct absorption spectra (Fig. 2B) ranging from full green (CA_1_)– to full blue–acclimated state (CA_6_) with four intermediate acclimation states (CA_2_ to CA_5_) representing in-between absorption characteristics (Fig. 2B). We tested several numbers of intermediate states and determined that four was optimal as experimental data showed the existence of at least four states with distinct absorption properties and using a larger number did not yield substantial alterations in the results (fig. S1). The experimental data also suggested that using a linear interpolation between CA_1_ and CA_6_ was a reasonable method to estimate CA_2_ to CA_5_ in the model.

### Modeling *Synechococcus* PT biogeography

The plankton functional groups implemented in our Darwin setup are analogs of *Prochlorococcus*, *Synechococcus*, pico-eukaryotes, coccolithophores, diatoms, mixotrophs, diazotrophs, zooplankton, and heterotrophic bacteria. The biogeography of these groups emerges from the interactions of several factors such as physical transport, spectral light fields, multiple nutrient supply rates, competition for resources, and grazing. Distribution patterns are consistent with previous versions of the model and compare well to observations (*29*, *30*) (fig. S2).

**Fig. 3.**
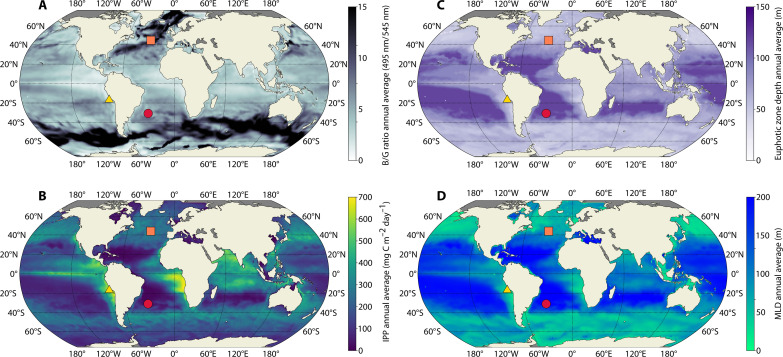
Annual patterns of modeled oceanographic variables. The global patterns of (**A**) MLD, (**B**) integrated primary production (IPP), (**C**) euphotic zone depth, and (**D**) average 0- to 100-m blue light–to–green light ratio that emerged in the simulated ocean. The yellow triangle, red circle, and orange square indicate the position of the example upwelling, subtropical, and temperate locations discussed in the text (Figs. 4 and 5).

Mixed layer depths (MLDs) are deep at high latitudes and shallow in equatorial and coastal upwelling regions (Fig. 3A). Nutrient-rich regions, such as upwelling areas, exhibit large primary production levels (Fig. 3, A and B). Consequently, these areas display shallower euphotic zone depths [defined as 1% of surface photosynthetically available radiation (PAR)] (Fig. 3C) and a higher prevalence of green light relative to blue light, as indicated by the average blue-to-green ratio (B/G) within the upper 100 m of the water column (Fig. 3D). The B/G here represents the ratio of available light intensity (scalar irradiance) at 495 to 545 nm, corresponding to the absorption peaks of PUB and PEB, as a proxy for quantifying the relative availability of blue and green light. Nutrient-depleted regions such as the center of the subtropical gyres typically feature lower primary production values, deeper euphotic zones, and higher B/G. The B/G generally increases with depth because the bluer wavelengths persist deeper in the water column than longer wavelengths because of the water optical properties (*36*).

**Fig. 4.**
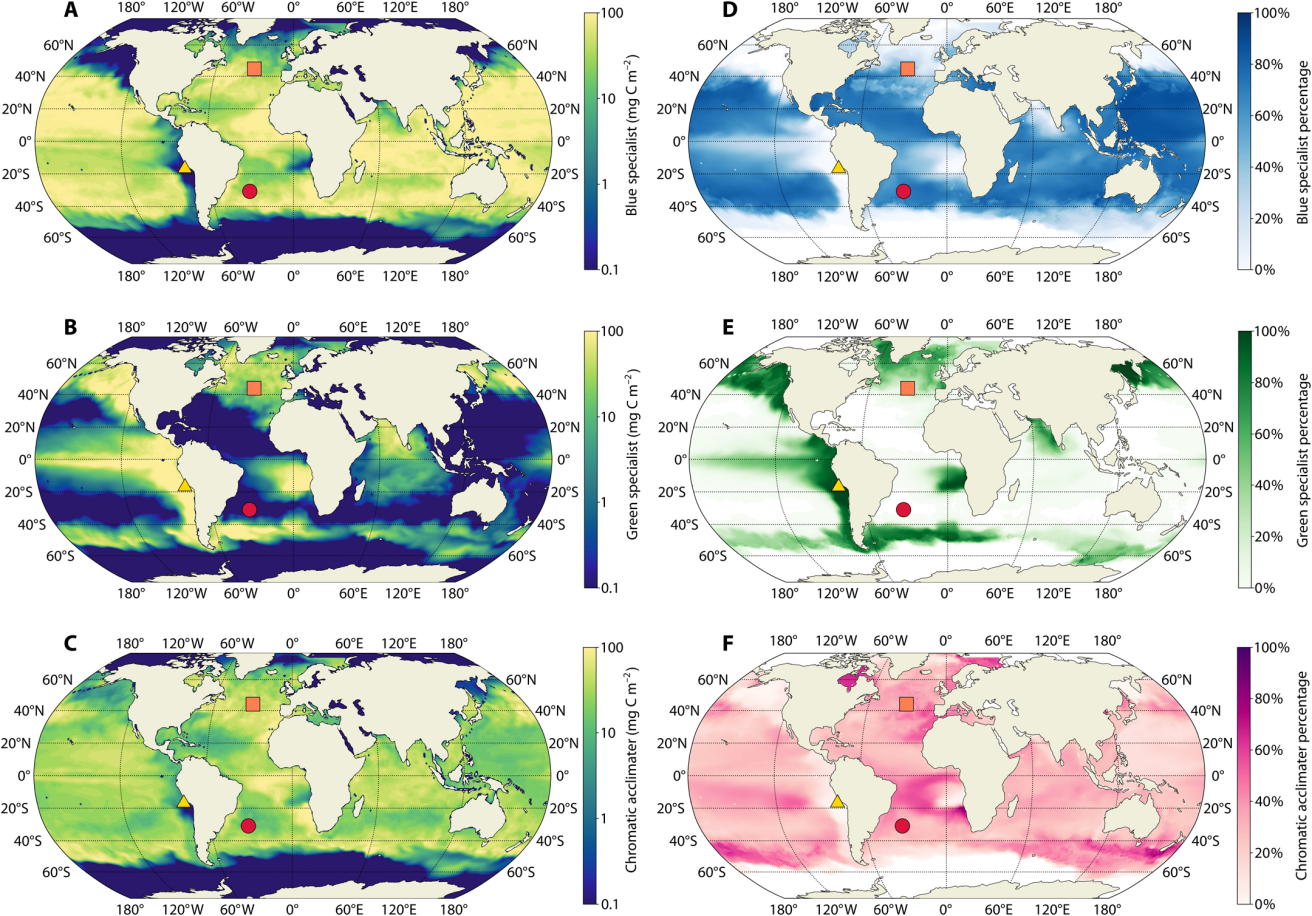
Biogeography of simulated *Synechococcus* PTs. (**A** to **C**) Annual average biomass distribution integrated from the surface to 200 m (mg C m^−2^) of (A) the BS, (B) GS, and (C) CA. (**D** to **F**) Annual average percentage contribution relative to the total *Synechococcus* biomass integrated within the first 200 m for the BS (D), GS (E), and CA (F). The three markers on the maps (yellow triangle, red circle, and orange square) indicate the position of the example upwelling, subtropical, and temperate locations discussed in the text (Figs. 4 and 5).

The inclusion of three distinct *Synechococcus* PTs enabled the first estimation of the biogeography associated with their absorption properties. In the simulation, the BS, GS, and CA accounted for ~52, 18, and 30% of the annual global *Synechococcus* biomass integrated over 200 m (Fig. 4 and movie S1). The BS is widespread and particularly abundant in oligotrophic regions such as subtropical gyres (Fig. 4A), consistent with observations (*14*). Conversely, the GS dominates in upwelling and eastern ocean margins (Fig. 4B) usually associated with shallower euphotic depth and high productivity (*18*, *37*, *38*) (Fig. 3, B and C). CA is ubiquitous within the *Synechococcus* niche in the modeled ocean (Fig. 4C). They represent most *Synechococcus* biomass in transitional zones between BS-dominated and GS-dominated regions. The CA widespread distribution displays a larger overlap with the BS compared to GS (Fig. 4C).

The consistent *Synechococcus* absence from high-latitude seas, like the Southern Ocean, was not due to physiological constraints, such as lower thermal tolerance, as no temperature limits were imposed on the different plankton types in the model. Instead, it was primarily attributed to their relatively low growth rate (compared to, e.g., diatoms) along with factors such as apparent competition with heterotrophic bacteria and similarly sized, faster-growing eukaryotes (*31*).

### Comparison of the Darwin model with the *Tara* Oceans dataset

Analyses of the *Tara* Oceans metagenomes provided the first global-scale picture of marine *Synechococcus* PT distribution (*13*). Although these data are relatively sparse, lack seasonal information, and provide relative gene abundance instead of biomass, they remain the most comprehensive information available to date on the biogeography of *Synechococcus* PTs. The data notably revealed the widespread distribution of CA on a global scale (Fig. 1). To evaluate the agreement between the model and the *Tara* Oceans dataset and to overcome the differences in data typology, we assessed how often the model and in situ data identified the same dominant PT. In addition, we quantified this agreement using the Matthews correlation coefficient (*39*). The value of this coefficient ranges from −1 to 1, where 0 indicates no better agreement than random chance, 1 signifies perfect agreement (i.e., the two datasets are identical), and negative values indicate agreement that is worse than random chance (see Materials and Methods for further information). We compared the model output to available *Tara* Oceans data at the surface and deep chlorophyll maximum depth. The model correctly predicted the dominant PT for 66% of the samples with a Matthews correlation coefficient of 0.44 (see Supplementary section 5 for further details). Notably, the Darwin model was able to reproduce the dominance of the GS in the Arabian Sea and eastern equatorial Pacific (*37*, *38*). The model also captured the large percentage of BS in oligotrophic regions such as subtropical gyres and the Mediterranean Sea (*14*). The CA relative abundance was well represented in temperate waters such as the Mediterranean Sea and North and South Atlantic as well as in the South Indian and Pacific Ocean, where it most often coexisted with BS, consistent with observations (*13*). The model did not correctly predict the dominance of GS in the southern Caribbean Sea. The main discrepancies between model output and *Tara* Oceans data could in part be attributed to the coarse resolution of the Darwin model that does not capture some of the finer coastal dynamics.

### Regional and seasonal variations in abundance of *Synechococcus* PTs

**Fig. 5.**
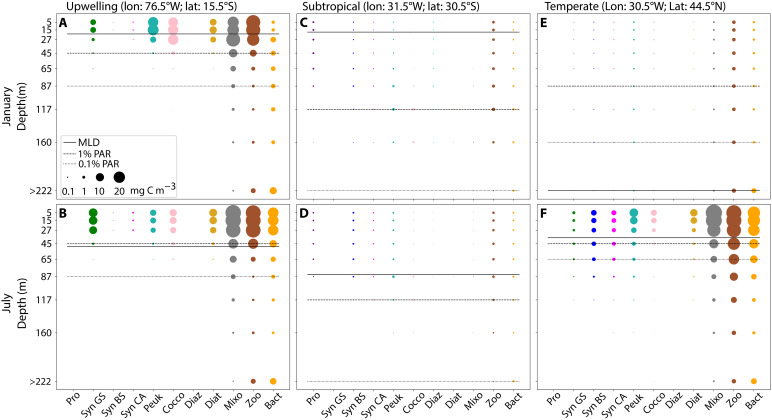
Spatiotemporal variability of the modeled planktonic community. (**A** to **F**) Each column in the figure corresponds to the January and July biomass distribution of plankton functional groups (mg C m^−3^) within the water column for a different selected location. The size of the bubbles is proportional to the biomass of the respective groups. The solid horizontal line represents the MLD, while the depths at which light intensity corresponds to 1 and 0.1% of surface PAR are indicated by the dashed and dotted lines, respectively.

To illustrate the vertical and temporal dynamics of *Synechococcus* PTs in more detail, we selected upwelling, subtropical, and temperate locations (Figs. 3 and 4) as examples of contrasting environments (Fig. 5).

The example upwelling location displayed a moderate degree of seasonality with consistently shallow MLD and euphotic zone (Fig. 5, A and B). Because of the continuous upwelling of nutrients, this regime was associated with high planktonic biomass levels leading to shallower euphotic zones and greener light throughout the year (Fig. 3). Biomass was confined to shallower depths compared to the other locations. In January, phytoplankton biomass was dominated by pico-eukaryote and coccolithophore analogs followed by *Synechococcus* and diatoms. In July, *Synechococcus* was abundant in the mixed layer, along with diatoms, coccolithophores, and pico-eukaryotes. *Synechococcus* biomass was dominated by GS in the water column throughout the year at the upwelling location (≥95%). CA accounted for the remaining small percentage of *Synechococcus* biomass.

The example oligotrophic subtropical location (Fig. 5, C and D) exhibited low seasonality in terms of MLD and minimal variation in the light field, biomass levels, and planktonic community composition. The biomass of autotrophs was extremely low because of nutrient paucity and predominantly composed of pico-phytoplankton all year round. Consequently, light penetrated deeply into the water column and the available light field was dominated by blue wavelengths (Fig. 3D). *Synechococcus* exhibited low levels of biomass mostly composed of BS (~70%) and CA making up the remaining 30%. The vertical distribution of the BS and CA was similar and homogeneously distributed within the mixed layer.

The temperate location (Fig. 5, E and F) was affected by strong seasonality. In January, the reduced light levels coupled to a deep MLD resulted in reduced phytoplankton production. Accordingly, the overall planktonic community biomass was low and the euphotic zone was deep. The bulk *Synechococcus* biomass integrated over 200 m was low, homogeneously distributed, and mostly composed of BS (~43%) and CA (~40%). In July (Fig. 4F), the higher surface-incident irradiance, stronger water column stratification, and higher phytoplankton biomass led to a shallower mixed layer and euphotic zone. Pico-eukaryotes, *Synechococcus*, and diatoms were the most abundant phytoplankton groups. The higher primary production also sustained increased biomass levels of mixotrophs, zooplankton, and heterotrophic bacteria. *Synechococcus* depth-integrated biomass was roughly 30 times higher in July compared to January, yet the dominant PT remained the BS (~50%), closely followed by CA (~40%). The vertical distribution of *Synechococcus* biomass showed that the BS and CA colonize deeper layers than GS.

## DISCUSSION

The biological and ecological importance of *Synechococcus* PTs has received growing attention from the scientific community over the past decade (*6*, *13*, *22*, *40*, *41*) because of the wide spatial distribution and the important role in global primary production of this cyanobacterium. Incorporating *Synechococcus* BS, GS, and CA into a complex 3D ecosystem model notably enhanced the level of processes taken into account compared to previous studies (*42*, *43*). The Darwin model simulates the global ocean, accounting for numerous factors, such as the passive transport of plankton groups, competition for vital resources such as light and nutrients, and grazing. It also resolves feedback between the planktonic community, other optically active constituents, and the light environment. These elements work synergistically to shape the modeled ecosystem. The customized model used in this work enabled us to estimate the global distribution of three different *Synechococcus* PTs, to explore their spatiotemporal dynamics, and lastly, to investigate how different absorption characteristics, including chromatic acclimation, contributed in shaping global phytoplankton biogeography. *Synechococcus*’ ability to efficiently harvest light across different wavelengths, combined with its small size and low nutrient requirements, allows for widespread global distribution in the model, as in reality (*15*). This cyanobacterium has a substantial impact on carbon sequestration and food web structuring, supporting trophic cascades that extend from microbial grazers to commercially important fish species and pelagic predators (*15*, *29*, *44*, *45*). What sets the distribution of *Synechococcus* is thus important for understanding ocean food webs and carbon cycling. Our study has showcased the importance of the PTs and chromatic acclimation, helping set the distribution of this cyanobacterium. This is a crucial step toward elucidating potential intricate interactions and cascading effects, such as those driven by climate change–induced shifts in ocean color, that will define marine ecosystem dynamics in a rapidly changing world.

It is worth stressing that the features driving the emergent biogeography are not only determined by the different light harvesting capabilities but, rather, they emerge as outcomes of a complex web of interactions between biotic and abiotic ecosystem components (*29*–*31*).

The quantitative comparison analysis conducted to evaluate the agreement between the Darwin model and discrete observations from the *Tara* Oceans transect demonstrates the model’s capacity to capture broad spatial and temporal dynamics of global ocean patterns rather than replicating precise local observations. This agreement is particularly noteworthy because the model was parameterized on the basis of targeted lab experiments to test ecological outcomes of a well-resolved physiological process without tuning parameters to fit field data. By focusing on the dominant *Synechococcus* PT, we bridged the mismatch between the model’s carbon-based biomass output and the *Tara* Oceans dataset’s relative gene abundance, enabling a categorical comparison. Furthermore, the agreement between the model’s PT distribution and global patterns established in the literature (*13*, *41*, *46*) underscores the robustness of this approach, which is grounded in experimentally derived data informed by decades of *Synechococcus* research (see Supplementary section 1).

*Synechococcus* PTs, especially BS and GS (Fig. 4, D and E), show strong niche complementarity reflecting their ability to harvest energy from different spectral regions of the light field. The CA widespread distribution (Fig. 4C) can be ascribed to their ability to adjust their PUB/PEB ratio, enabling them to compete or coexist under certain conditions, with the specialists. Notably, the CA exhibits an asymmetry in its ability to regulate the PUB/PEB ratio. While this PT can align this ratio closely with the BS spectrum in blue light, it is unable to reach as low PUB/PEB ratio as the GS in green light (*21*) (Fig. 2A). In agreement with this asymmetry, we find that the CA and BS analogs share a large portion of their niches, while the CA biogeography is more extended than the BS, especially toward upwelling and coastal areas (Fig. 4, A and C). The notably different absorption properties between GS and the other two PTs caused a lower overlap of potential and realized niches.

To gather further insights on PT competition and investigate the advantage conferred by chromatic acclimation, we explored the link between PT distribution, CA phenotype, and light field features under different environmental conditions. The CA has the potential to more effectively exploit the vertical variability of the light field by using intermediate acclimation states. Specifically, we consider the “best acclimation state” as the one able to harvest the largest amount of energy from the available light field or, in other words, the one with the photosynthetic absorption spectrum best matching the available light color. The CA in the model may not be in the best acclimation state at any given time, but it is useful to assess the optimum state that the CA is striving to achieve. We compare this to B/G as a proxy for quantifying the relative availability of blue and green light, specifically at the wavelengths most efficiently absorbed by the PEB and PUB phycobilin pigments that the CA is switching between. The rate of increase and the magnitude of the B/G through the water column depend on environmental conditions and optically active constituents (*36*).

**Fig. 6.**
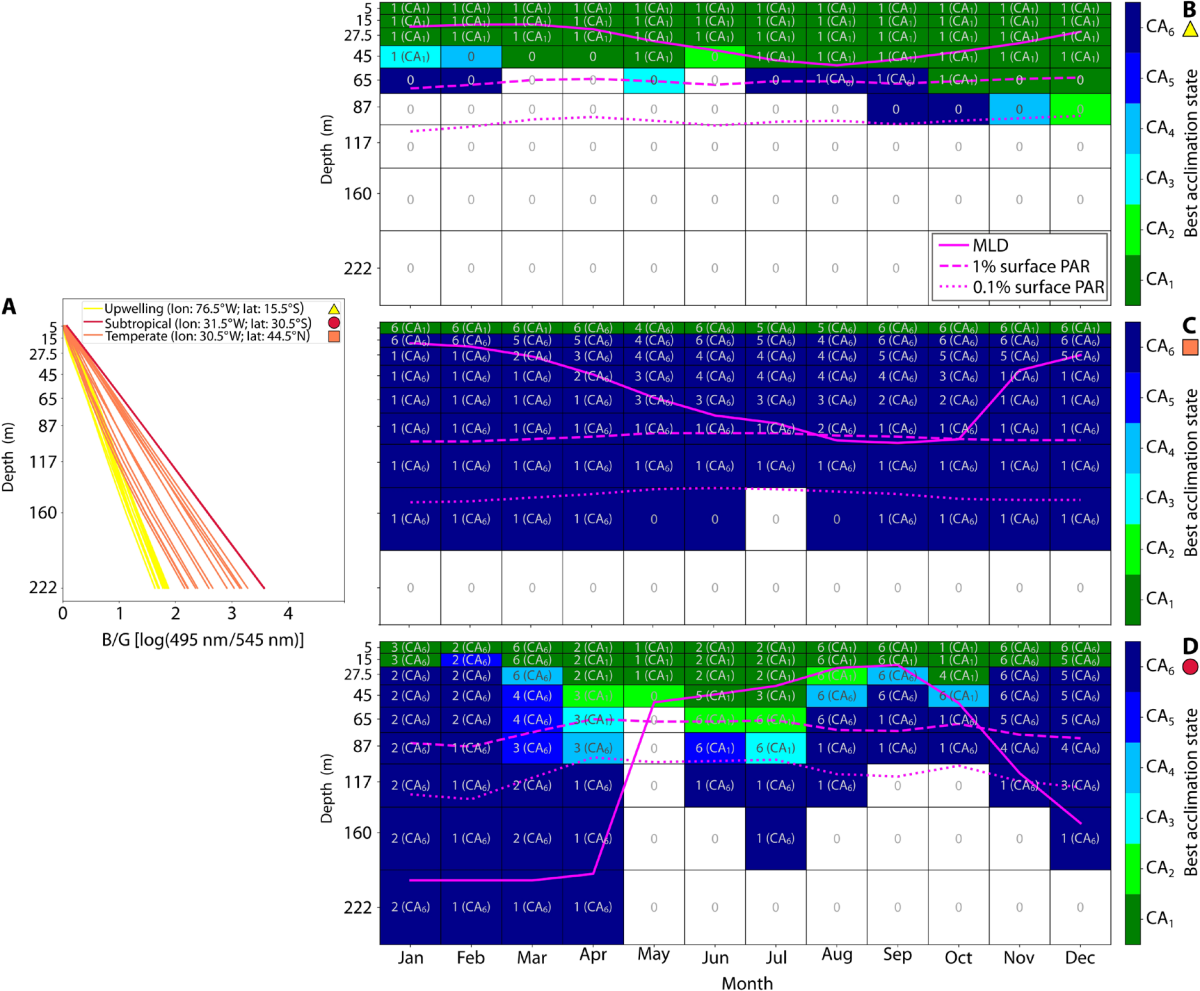
Plasticity of CA absorption properties. (**A**) Monthly vertical profiles of the blue light–to–green light ratio for each regime. The ratio is computed from the modeled light field using the available light at 495 and 545 nm, which correspond to the absorption peaks of PUB and PEB, respectively. (**B** to **D**) Monthly vertical profiles of the acclimation states for the upwelling (B), subtropical (C), and temperate (D) regimes. Color shading represents the best acclimation state: the state most efficient in harvesting the available light for a given portion of the water column and month of the year (i.e., representing the acclimation target for all CAs in different states). The number outside the brackets in each box shows how many acclimation states coexist in the depth bin, while the number inside the brackets indicates the dominant acclimation state in terms of biomass. See fig. S7 for a more detailed breakdown of the percentage of each acclimation state. The grid lines represent the time and depth resolution of the model output. The thick solid line represents the MLD, while the depths at which light intensity corresponds to 1 and 0.1% of surface PAR are indicated by the dashed and dotted lines, respectively.

The example coastal upwelling location displayed a constitutively green available light field (Fig. 3D), leading to the dominance of GS in these regions, while CA accounts for a small percentage of *Synechococcus* biomass and BS is outcompeted (Figs. 4 and 5, A and B). The temporal analysis of the B/G monthly depth profiles (Fig. 6A) shows a gradual increase with depth of this ratio and lower values compared to temperate and subtropical regions. In this scenario, the most green-acclimated state (CA_1_) emerged as the best acclimation state for the CA in the whole water column all year round (Fig. 6B and fig. S7). The negligible contribution of the acclimation states CA_2_ to CA_6_, coupled with the suboptimal ability to absorb green wavelengths because of the physiological limits of chromatic acclimation, is arguably the predominant reason why CA could not compete with GS in this region (Fig. 5, A and B, and 6A). We found that CA biomass was only maintained in this location because of advection from adjacent bluer regions (Supplemental section 5).

Oligotrophic regions, such as the example subtropical location, are characterized by a lack of seasonality in the light field, which is dominated by blue wavelengths (Fig. 3D). All monthly B/G profiles almost overlap, showing a rapid increase in the ratio with depth and high values throughout the water column (red lines in Fig. 6A). Under these conditions, the BS was the best adapted, followed by the CA. The GS analog is completely absent in this location because of its inability to compete for the prevalent wavelengths. The best acclimation state was consistent throughout the year (Figs. 5, C and D, and 6C) as a result of weak seasonality in the light field. The distinctive feature in this area is related to CA_1_ being the best adapted in the first layer of the model, where green light is still available before being absorbed by optically active constituents, yet the CA_6_ is overall the best adapted from subsurface downward. This does not imply that all the CA biomass is exclusively found in these two states but only indicates that these acclimation states are the most effective in exploiting the available light field at a certain depth, thus determining the direction of the acclimation process (fig. S7). The abrupt transition of the best acclimation state from CA_1_ to CA_6_ seems to grant access to abundant and underexploited green wavelengths near the surface while also enabling the utilization of dominant blue wavelengths in subsurface layers. As a result, switching between the two specialist-like phenotypes seems to be the preferred strategy under these conditions as the plankton are constantly moving through the mixed layer (Fig. 6C and fig. S7).

In regions of strong seasonality, there is a marked variability in the light field over the course of the year (e.g., orange square, Fig. 3D). The CA accounted for a large share of *Synechococcus* biomass in these regions, suggesting that it can capitalize on the temporally changing conditions. The seasonality can result in the coexistence of *Synechococcus* PTs through depth and time. The GS is less competitive in deeper mixed layers where the average light field is relatively bluer (Fig. 5B). This observation underscores how the deeper penetration of bluer wavelengths, in conjunction with distinct absorption properties, plays a crucial role in determining PT spatial distribution.

The monthly B/G profiles of this region (orange lines, Fig. 6A) display an increase in the rate of change within the water column that ranges from the slow one of the upwelling location to almost the fast increment of the subtropics. The dominance of gradual transition from green to blue wavelengths through depth created multiple spectral niches throughout the water column (and, hence, multiple best acclimation states). Under these conditions, the advantage conferred by chromatic acclimation is not simply from switching between the two specialist-like phenotypes but stems from the presence of intermediate acclimation states that are more efficient than the specialists in exploiting the available light (Fig. 6D and fig. S7), thus potentially explaining the large biomass of chromatic acclimation in temperate regions (Fig. 4, D and F).

Through this analysis of the light field, it becomes evident that the advantage conferred by chromatic acclimation consists in both the ability to mimic a specialist-like phenotype and the exploitation of specific blue-green light niches made possible by intermediate acclimation states. The CA preferentially adopts a specialist-like phenotype in regions with low light field seasonality while using both strategies in areas with high variability.

We additionally found that explicitly resolving the different light absorption properties of several *Synechococcus* PTs in the model resulted in an expansion of realized *Synechococcus* niche and a higher average biomass (Supplemental section 5 and table S1) when compared to simulations with only one *Synechococcus* PT. We also found that excluding the CA and only including the specialists led to substantial changes in *Synechococcus* abundance and biogeography. Biomass decreased over 58% of the ocean without the CA (Supplemental section 3 and fig. S3A), with temporally as much as 38% lower *Synechococcus* biomass in some regions (fig. S3, B to G, and table S1). These findings suggest that not only the diversity of pigment types but also, particularly, chromatic acclimation conferred an overall fitness advantage to *Synechococcus* through enhanced exploitation of the light resource. This also underscores the importance of considering diversity in ecosystem models.

The global biogeography of *Synechococcus* is set by bottom-up (e.g., resource acquisition) and top-down (e.g., grazing) processes within a dynamically moving and temporally varying environment. Here, we have shown that being able to better harness light energy by using several pigment combinations provides an advantage to *Synechococcus*. In particular, we demonstrate that having a PT that can modify its pigment composition provides a competitive advantage where organisms are exposed to varying spectral light quality. Our findings suggest that this advantage is twofold: the ability to reversibly mimic specialists’ absorption characteristics is beneficial when constantly mixed between the surface greener waters and the deep bluer waters; in addition, optimizing light harvesting at intermediate acclimation states provides an advantage in highly seasonal and variable environments. These advantages led *Synechococcus* to increase its range and biomass in the model. Such adaptations are particularly important in aquatic environments, especially in the ocean, where the variability in optical conditions is more readily found than, for example, in terrestrial plant environments. In marine ecosystems, phytoplankton are transported vertically through a highly variable light spectrum and dispersed to/from contrasting hydrographic regimes via lateral advection. Thus, this work not only enhances our understanding of the ecological dynamics involving *Synechococcus* and oceanic phytoplankton biogeography but also underscores the importance of chromatic acclimation as an evolutionary strategy more generally. Furthermore, we highlight the importance of the complex interplay between physics, biogeochemistry, and ecosystem in the advantages of this trait.

The importance of enhancing our understanding of PT distributions, interactions, and the role of chromatic acclimation is even more important in the context of climate change, which is predicted to cause a shift in the planktonic community composition and ocean color (*47*–*49*).

## MATERIALS AND METHODS

### Biogeochemical ecosystem model

The 3D biogeochemical ecosystem model used in this work (Darwin model) resolves the cycling of carbon, nitrogen, phosphorus, silica, and iron through biotic and abiotic pools. Biogeochemical and biological components are transported within a general circulation model (MITgcm) (*50*) with a 1° by 1° horizontal resolution and 23 vertical levels ranging from 10 m in the surface to 500 m at depth. The living component of the modeled ecosystem follows Follett *et al.* (*30*) and consists in several plankton functional-type analogs of *Prochlorococcus*, *Synechococcus*, and a range of size classes of pico-eukaryotes, coccolithophores, diatoms, mixotrophs, diazotrophs, zooplankton, and heterotrophic bacteria. The size of the different plankton types influences physiological rates and grazing. The susceptibility to grazing follows a fixed predator-prey size ratio (*29*, *30*, *51*). The biomass of each plankton functional group was initialized at a low level to prevent an early artificial dominance of any specific analog. The model was run for 10 years with a 3-hour time step. The global planktonic community composition emerges from the interactions between physical, chemical, and biological components of the modeled ecosystem.

The Darwin model includes optical properties of seawater, detritus, colored dissolved organic matter, and plankton. The radiative transfer component is based on the Ocean–Atmosphere Spectral Irradiance Model (*52*) with modifications (*29*). The model uses the total light absorption spectrum of the different optical components to compute light attenuation. Phytoplankton growth is computed using the photosynthetic absorption spectra estimated using the pigment reconstruction technique (*11*, *53*). Here, we used a 5-nm resolution for the radiative transfer model and the optical properties of seawater, detritus, colored dissolved organic matter, and plankton. This is a higher spectral resolution than the 25 nm used in previous versions of the Darwin model (*29*, *30*) and enabled an improved representation of *Synechococcus* PT absorption properties. Using a 5-nm resolution allowed the model to catch the absorption peak of PUB and PEB, which was critical to investigate the competition dynamics between PTs and the advantage conferred by chromatic acclimation.

The choice of the Darwin model to address the importance of chromatic acclimation was driven by several motivations. First of all, this model resolves spectrally the optical properties of phytoplankton and other optically active constituents in a simulation of the global ocean. The Darwin model also takes into account the interactions between the plankton analogs, their competition for resources, and their losses from grazers within a simulated dynamic physiochemical environment, which is essential for assessing the competitive advantage conferred by chromatic acclimation.

### Modeling chromatic acclimation

The implementation of chromatic acclimation into the Darwin model was based on experimental data. The blue-green acclimation time in *Synechococcus* is irradiance dependent and ranges from 6 to 3 days depending on whether the cells are exposed to low (~20 μmol photons m^−2^ s^−1^) or high (~75 μmol photons m^−2^ s^−1^) light conditions (*21*, *35*).

A set of experiments was carried out to study the details of the acclimation process from full blue– to full green–acclimated *Synechococcus* CA. These experiments complemented previous information about intermediate acclimation states being linked to a gradual transition in PUB/PEB ratios (*35*) (fig. S1).

We conducted a series of sensitivity studies testing a range of 3 to 12 acclimation states resolved in the model. We determined that six acclimation states (CA_1_ to CA_6_) was the minimum number that allowed us to simulate a gradual and physiologically sound acclimation process between green and blue light while avoiding the introduction of unnecessary complexity into the model. The full blue–acclimated CA (CA_6_) shows an estimated PUB/PEB ratio similar to the one exhibited by the BS (PUB-rich: PUB/PEB ~1.4), while the full green–acclimated CA (CA_1_) displays a larger PUB/PEB (~0.67) with respect to the GS (PEB-rich: PUB/PEB ~0.4) (*3*, *13*, *21*).

The pigment reconstruction technique used to determine the photosynthetic absorption spectra consists of fitting the absorption of specific pigments or pigment complexes to the measured spectra (*11*, *53*). Total absorption and photosynthetic absorption refer to absorption by all pigments and only pigments used for photosynthesis, respectively. The former was used in the model to compute light attenuation in the water column, while the latter was used to estimate phytoplankton growth rates. However, the photosynthetic pigment reconstruction was not possible for the acclimation states CA_1_ to CA_5_ as there was not enough information about phycobilisomes in intermediate acclimation states. For this reason, the absorption spectra for the acclimation states CA_1_ to CA_5_ were created by interpolating between the spectra of CA_6_ and the average spectra of the GS, taking into account the lower limit of PUB/PEB for CA_1_ (Fig. 2B).

All the acclimation states (CA_1_ to CA_6_) were implemented into the model as analogs of the CA. The model simulated their acclimation ability by redistributing biomass from all acclimation states toward the one that could most efficiently capture light from the available light field. Biomass movement was restricted to neighboring acclimation states, replicating a gradual physiological transition. Furthermore, this biomass movement was designed to be time and light intensity dependent, aligning with the established physiological understanding of *Synechococcus* chromatic acclimation.

We performed several model simulations to obtain and support the key results presented in this work (full details provided in the Supplementary Materials). The main simulation included the three different *Synechococcus* PTs (BS, GS, and CA) in the Darwin setup. We then conducted simulations that included only one (BS or GS) or two *Synechococcus* PTs (BS and GS) and compared them to the “main” simulation to assess the impact of increased diversity and the presence of chromatic acclimation on *Synechococcus* range and biomass. Last, we conducted simulations with lateral advection of plankton turned off (one with all three PTs included and one with only the two specialists) to test the hypothesis that moving through different water masses was advantageous for *Synechococcus* when chromatic acclimation was taken into account.

### Model validation

To assess the agreement between model predictions and the *Tara* Oceans dataset, we used the Matthews correlation coefficient to compare the dominant PT predicted by the Darwin model with the dominant PT observed at *Tara* Oceans sampling sites. The selection of this coefficient, coupled with the focus on the dominant PT, allowed us to overcome the mismatch between the Darwin model output (biomass in carbon) and the *Tara* Oceans data (relative gene abundance), allowing for a comparison between categorical data. The Matthews correlation coefficient quantifies the agreement beyond chance by incorporating both observed and expected agreement if classifications were random [values can be interpreted similarly to Pearson’s correlation coefficient; (*54*)]. The coefficient value ranges from −1 to 1, where 1 indicates perfect agreement between the model and observations, −1 is complete disagreement, and 0 represents agreement occurring purely by chance. This comparison generated a confusion matrix, which enabled the calculation of the coefficient, providing an objective measure of how accurately the model reflects observed PT distribution patterns. The Matthews correlation coefficient considers all elements of the confusion matrix. This makes the coefficient particularly robust for scenarios where class distributions are skewed, as it avoids inflated performance metrics caused by majority class bias (see Supplementary section 5 for further details). Matthews correlation coefficient values were interpreted within the context of global ecological modeling, acknowledging the inherent scale mismatch between the model’s broad, long-term predictions and the localized, temporally distinct *Tara* Oceans observations.

## Supplementary Materials

### The PDF file includes:

Supplementary Text Figs. S1 to S7 Table S1 Legend for movie S1 References
