# Supplementary material for: Chromatic acclimation shapes phytoplankton biogeography
Source: Sci Adv. 2025 Feb 19;11(8):eadr9609. doi: 10.1126/sciadv.adr9609 (PMC11838011; doi:10.1126/sciadv.adr9609)
Supplement: Supplementary file 1 — Supplementary Text Figs. S1 to S7 Table S1 Legend for movie S1 References [file sciadv.adr9609_sm.pdf]

Science Advances
AAAS

Supplementary Materials for

Chromatic acclimation shapes phytoplankton biogeography

Francesco Mattei *et al.*

Corresponding author: Francesco Mattei, francesco.mattei@imev-mer.fr

Sci. Adv. **11**, eadr9609 (2025)
DOI: 10.1126/sciadv.adr9609

The PDF file includes:

Supplementary Text
Figs. S1 to S7
Table S1
Legend for movie S1
References

Other Supplementary Material for this manuscript includes the following:

Movie S1

Supplementary Text

Supplemental section 1: Measurements and modeling of the absorption properties of the different *Synechococcus* pigment types

To incorporate analogues of *Synechococcus* blue specialist (BS), green specialist (GS), and chromatic acclimator (CA) into the Darwin model, we required distinct absorption spectra for each pigment type (PT). Initially, we gathered absorption spectra for BSs and GSs from the literature, accompanied by measurements of chlorophyll *a* (*32–34*). The chlorophyll *a* data were necessary for generating chlorophyll-normalized absorption spectra (m^2 mg chlorophyll^{-1}), a requirement for the Darwin model. Subsequently, we supplemented this disparate information with additional observations on absorption properties of a set of BS, GS, and CA strains grown in the same culture conditions. Twelve *Synechococcus* strains were retrieved from the Roscoff culture Collection (https://www.roscoff-culture-collection.org/) and cultivated at 25 °C and 75 µmol photons m^{-2} s^{-1}. The BS strains [CC9605 (RCC753), RS9902 (RCC2376), WH8102 (RCC539)] were grown under blue light and the GS strains [M16.1 (RCC791), RS9907 (RCC2382), WH7803 (RCC752)] under green light while CA strains [A15-62 (RCC2374), BL107 (RCC515), PROS-U-1 (RCC2369), RS9915 (RCC2553), RS9916 (RCC555), WH8020 (RCC751)] were grown under both light conditions to assess the light absorption differences relative to the two different acclimation states (see Dufour et al. 2024 (*35*) for further details).

Chlorophyll *a* concentration and light absorption spectra were concomitantly measured for all strains using high-performance liquid chromatography (HPLC) and spectrophotometry, respectively. Variable volumes of culture (11–45 mL) were filtered onto glass fiber filters (GF/F Whatman 25 mm), depending on cell concentration. Filters were successively flash frozen in liquid nitrogen and stored at -80°C until analysis at the SAPIGH HPLC analytical facility of the Institut de la Mer de Villefranche (IMEV), following the protocol described in Ras et al. (2008) (*55*). The absorption spectra of the phytoplankton cultures were determined using the same filters as used for the HPLC analyses, by scanning the filters with a Lambda 19 spectrophotometer (Perkin Elmer) equipped with an integrating sphere (Labsphere), with the filters placed inside the sphere. Absorption spectra were then corrected for the pathlength amplification and the units converted to m^{-1} using procedure described in Stramski et al. (2015) (*56*). Chlorophyll-specific absorption spectra (in units of m^2 (mg Chl)$^{-1}$) were obtained by normalizing the absorption spectra (m^{-1}) by the HPLC-determined chlorophyll *a* concentrations (mg Chl m^{-3}).

Spectra from literature and the additional BS and GS spectra were used to assess both intra and inter-PT variability. The 'total' absorption spectra (i.e. including photoprotective and photosynthetic pigments) of BS, GS, CA_1 and CA_6 model analogues was computed as the average of all the available spectra relative to that specific pigment type.

In addition, we analyzed absorption properties of fully blue light acclimated CA strains that were then shifted to green light, as described in Dufour et al (2024) (*35*). Samples were taken two to three times daily in order to get absorption spectra corresponding to intermediate acclimation states (see Dufour et al. 2024 (*35*) for further details). Figure S1 shows an example of absorption spectra taken at different times during the acclimation process of strain BL107 after being shifted from blue to green light. The intermediate absorption properties implemented for the intermediate chromatic acclimation states (CA_2-CA_5) in the model were consistent with those observed during the acclimation process (*21*, *35*) (fig. S1).

We explored various sensitivity scenarios, by testing from 3 to 12 acclimation states within the model, and found that 6 acclimation states represented the optimal compromise for simulating the

transition observed for CA strains absorption spectra from green to blue light while also avoiding unnecessary model complexity.

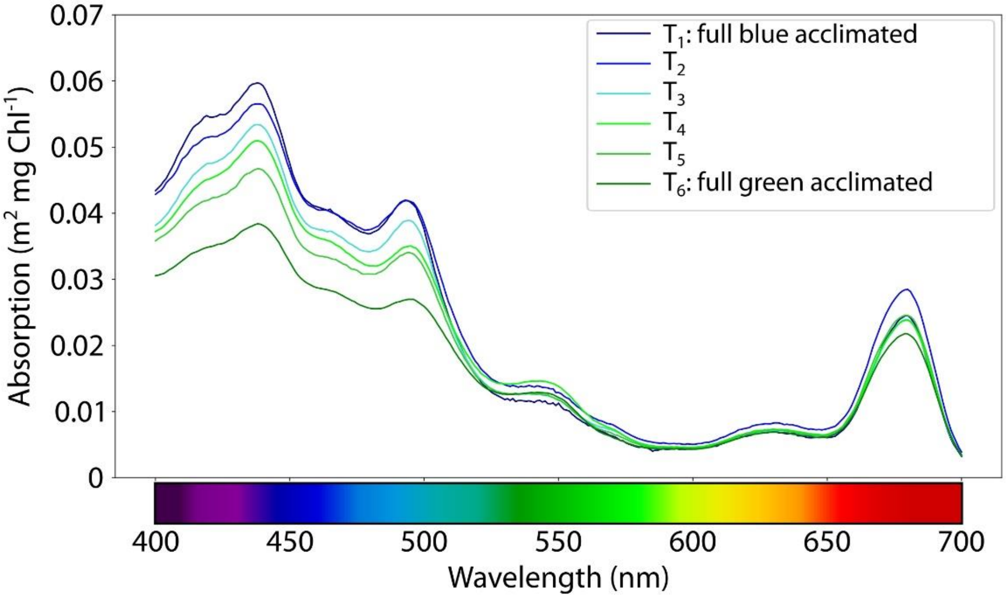

Fig. S1. Chromatic acclimator absorption spectra. The lines represent the total absorption spectra of a chromatic acclimator (strain BL107) taken at different times (T1-6) during the acclimation process from blue to green light. The spectra show a gradual absorption change from PUB to PEB-rich phycobilisomes during the acclimation process.

The chromatic acclimation timescale was defined in the model on the basis of experimental data (*21*, *35*). The acclimation time varied linearly from 6 to 3 days depending on the irradiance levels to which the CA analogues were exposed in the simulated Ocean. Low irradiance levels (~20 µmol photons m^{-2} s^{-1}) corresponded to 6 days for the acclimation from full green to full blue absorption features and vice versa (CA_1-CA_6), while under high irradiance levels the acclimation required 3 days.

The absorption spectra of photosynthetic pigments only were determined following a pigment reconstruction technique (see Hickman et al. 2010 (*11*) for details). In the model, the total light absorption alters the available light and absorption by photosynthetic pigments affects growth. Unfortunately, reconstructing the photosynthetic pigments for acclimation states $CA_{1\text{-}5}$ proved unfeasible due to insufficient data on phycobilisomes in these acclimatation states. Consequently, 'photosynthetic' absorption spectra (i.e., excluding photoprotective pigments) for $CA_{1\text{-}5}$ were generated by interpolating between CA_6 spectra and the average spectra of the GS, but setting the lower limit of PUB/PEB for CA_1 to values reported for green-acclimated CAs in the literature (*21*, *35*).

<u>Supplemental section 2: Impact of increasing diversity of *Synechococcus* and increased waveband resolution</u>

Two changes were necessary to the model configuration relative to Follett et al. (2023) (*30*) for this study: increasing i) the diversity of *Synechococcus* to account for different pigment types, and ii) spectral resolution from 25 nm to 5 nm. Supplemental figures 2 display the annual average

surface distribution of macro functional groups for Follet et al. (2023) (*30*) (first row), which were compared to MAREDAT dataset (*57*) (third row), and this work (second row). The distributions are similar and the differences can be mainly ascribed to the finer optical resolution used in our work.

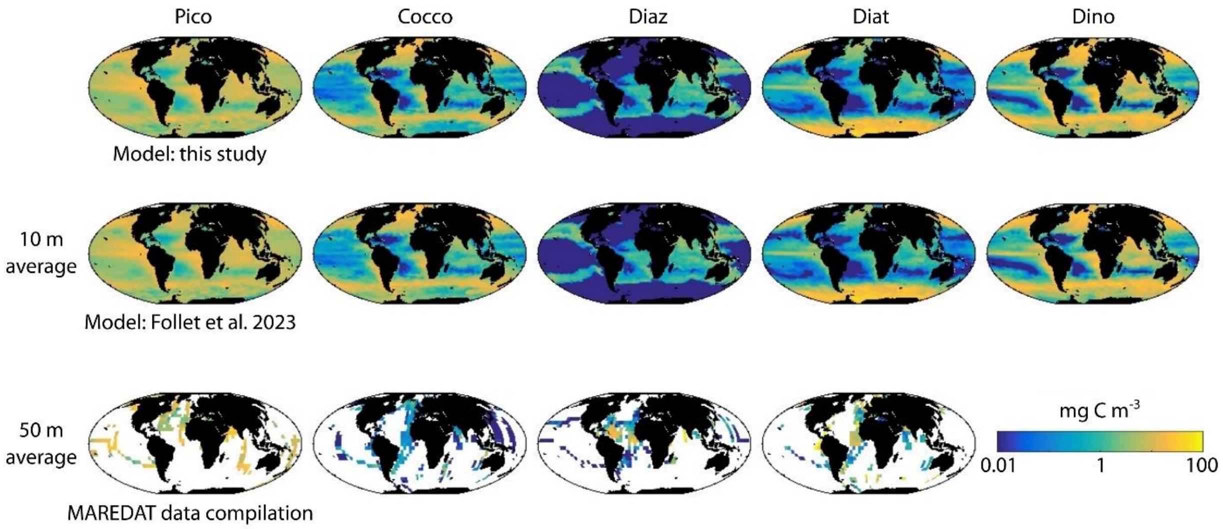

Fig. S2. Global surface distribution of phytoplankton macro functional types. The first and second rows illustrate the surface distribution of phytoplankton functional groups from the main simulation conducted in this work and Follet et al.'s study (2023) (*30*) respectively. The last row displays a compilation of MAREDAT (*57*) shipboard *in situ* data.

<u>Supplemental section 3: Model sensitivity experiments on impact of chromatic diversity within *Synechococcus*</u>

To evaluate the effect of chromatic acclimation, we compared the 'main' simulation and a simulation including only the two specialists (BS and GS) and not CA. When CA was not included, 58% of the simulated ocean exhibited lower total *Synechococcus* annual average biomass (fig. S3a, negative differences), whereas it was higher in only 28% of the ocean (fig. S3a, positive differences). Thus, including the CA resulted in 0.6% higher mean annual average *Synechococcus* biomass, with peaks reaching up to 9%, demonstrating the overall effectiveness of this trait. Monthly analysis revealed areas showing up to 38% more *Synechococcus* biomass and 30% more *Synechococcus* primary production when CA was included (fig. 3c, Table 1). The patchiness of positive and negative differences in certain areas was partially caused by a spatial rearrangement of nutrients. Specifically, the presence of CA led to a more efficient light field exploitation in certain regions which in turn caused a larger nutrient consumption and a consequent cascade effect on adjacent areas (*26*).

These findings suggest that chromatic acclimation conferred an overall fitness advantage to *Synechococcus* through enhanced exploitation of the light resource even when compared to an experiment including pigment diversity in the form of only the two specialists (BS and GS).

To evaluate the effects of an enhanced diversity of *Synechococcus* PTs, we compared the 'main' simulation with simulations with only one PT included (BS or GS). Comparison with simulations featuring only one specialist revealed even larger differences in overall *Synechococcus* biomass

and efficiencies in light field exploitation (Table S1). The GS-only simulation exhibited 3% lower average annual biomass with peaks up to 13%, while the BS-only simulation showed

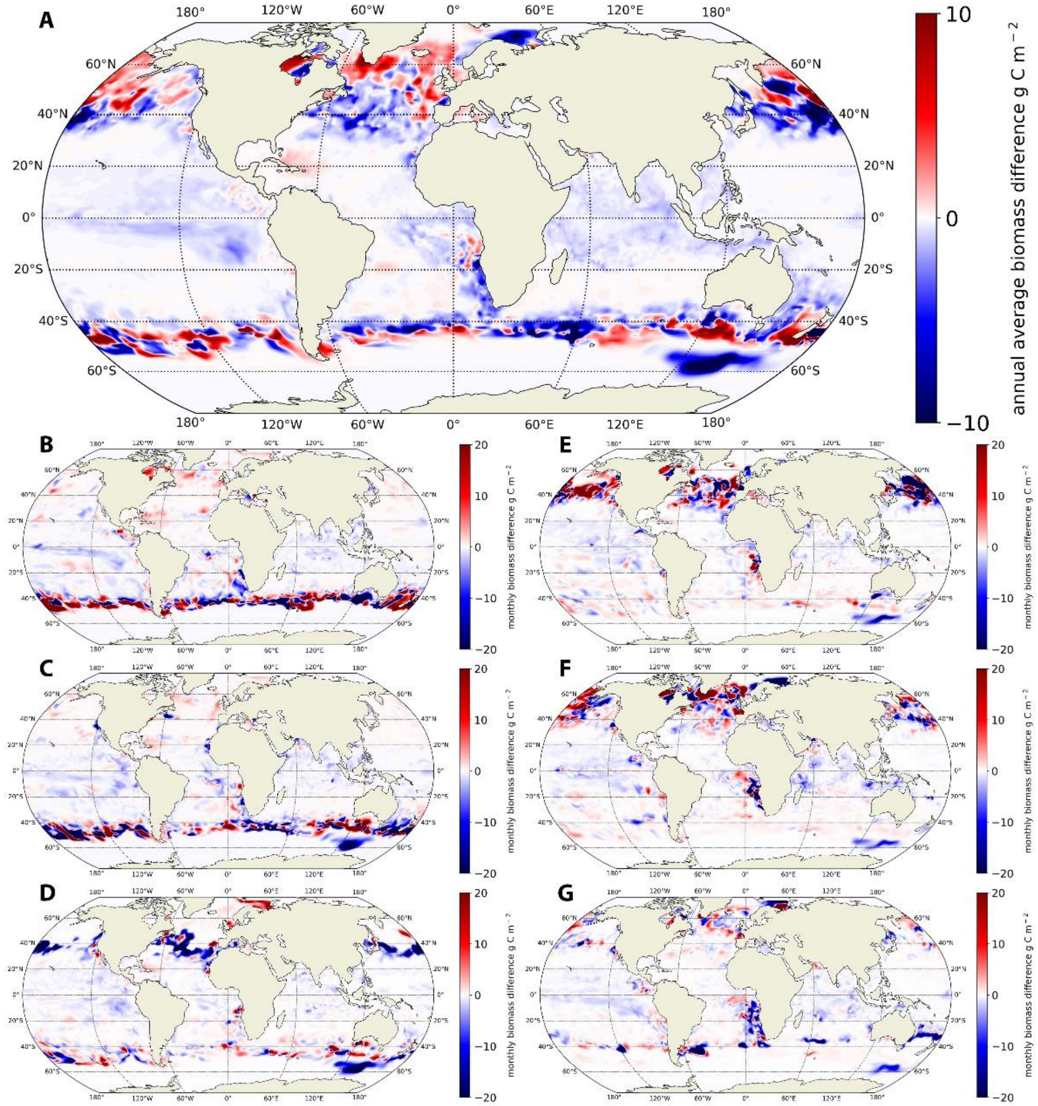

Fig. S3. Annual average *Synechococcus* biomass difference map. The maps show the differences in total *Synechococcus* biomass between the simulation including only the specialists (BS and GS) and the one implementing the chromatic acclimator (CA) alongside the specialists. Negative differences (in blue) depict a loss in *Synechococcus* biomass when chromatic acclimation is not implemented in the model, whereas positive differences (in red) denote a biomass gain in the simulation including only the specialists compared to the one with the chromatic acclimator. **A**, Map of the annual average biomass differences. Overall 58% of the simulated ocean shows lower biomass without chromatic acclimation, while only 28% display an increase of *Synechococcus* biomass. The global mean value of the annual average biomass increased by 0.6%, with certain areas experiencing notable increases of up to 9%. **B-D**, Monthly difference maps with and without chromatic acclimation. (**B** January; **C** March; **D** May; **E** July; **F** September; **G** November).

decreases of 2% and 19% when compared to the 'main' simulation (Table 1). Monthly data displayed lower *Synechococcus* biomass of up to 35% and 28% compared to the 'main' simulation for GS and BS-only simulations, respectively. These comparisons illustrate how pigment diversity and chromatic acclimation confer an advantage to the *Synechococcus* community by enabling a more efficient exploitation of the spatiotemporal variability in the light field characteristic of aquatic ecosystems, particularly the Ocean.

Supplemental section 4: Model sensitivity experiments on the importance of transport of plankton

The analysis of the modelled PTs distribution suggested that advection (lateral and vertical transport of water masses) could have been one of the sources of variability that provided an advantage to the CA. Being transported through different water masses associated with different light color niches could allow the CA to better exploit the light field by adjusting its pigmentation. We also hypothesized that the coexistence of CA and GS in specific regions, like the upwelling area investigated in detail in the main text, could result from the transport of CA from neighboring regions since its ability to compete for green wavelengths is limited by the physiological features of the chromatic acclimation (see main text Discussion). To test these hypotheses, we performed two additional simulations where advection for all plankton types was disabled, the first with all three Syn PTs (BS+GS+CA) and the second without CA (BS+GS only). With advection disabled, plankton analogues were only mixed vertically through the water column but were not allowed to move between neighborhood regions. Other modelled ecosystem components (e.g. nutrients and other optically active particles) were still able to move as in the 'main' simulation.

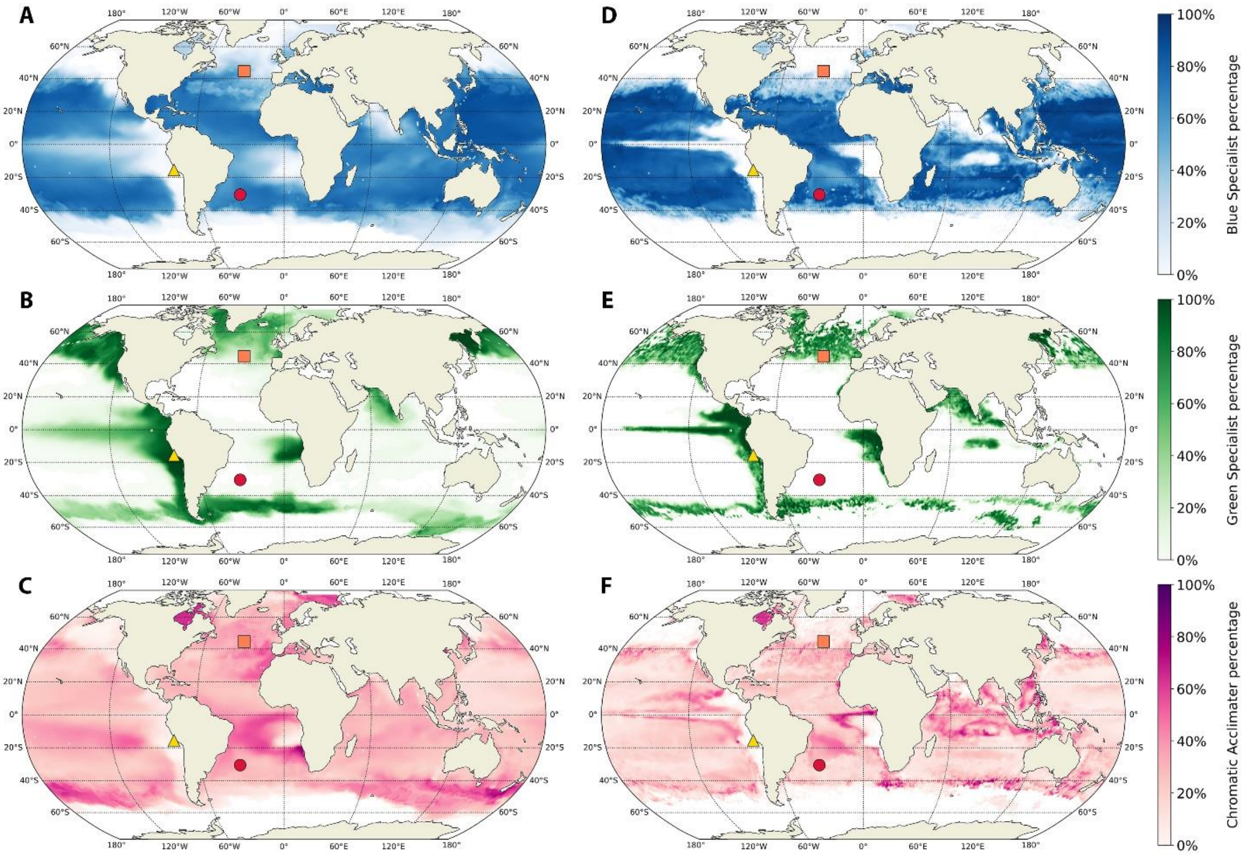

Fig. S4. Annual average biomass of *Synechococcus* pigment types as percentage of total *Synechococcus* biomass for the 'main' simulation (left column) and a simulation without advection (right column). In the simulation without advection, the proportions of BS and GS within the total *Synechococcus* biomass were 3.5% and 0.5% higher, respectively, while the percentage of CA was 4% lower. Without advection GS and CA did not coexist in the representative upwelling location (yellow triangle). The three markers on the maps (yellow triangle, red circle and orange square) indicate the position of the representative upwelling, subtropical and temperate locations discussed in the main text.

The first no-advection simulation (with all three Syn PTs), when compared to the 'main' standard simulation, showed that CA was the only PT negatively impacted when advection was disabled in terms of percentage of total Synechococcus biomass. In the 'main' simulation, BS, GS, and CA accounted for 52%, 18%, and 30% of total annual average Synechococcus biomass (integrated over 200 m), respectively, whereas without advection, they represented 55.5%, 18.5%, and 26% (fig. S4a,b,c vs S4d,e,f). Moreover, the small percentage of CA coexisting with GS in the representative upwelling location (yellow triangle fig. S4c) disappeared when advection was turned off (fig. S4c vs S4f).

The second no-advection simulation (with only BS+GS and no CA), when compared to the simulation with only BS+GS and advection turned on, further tested the hypothesis that CA benefited most from being advected through the ocean. This comparison revealed BS and GS constituted ~75% and ~25% of total *Synechococcus* in both scenarios (fig. S5). Thus, lateral advection does not lead to a competitive advantage to either BS or GS.

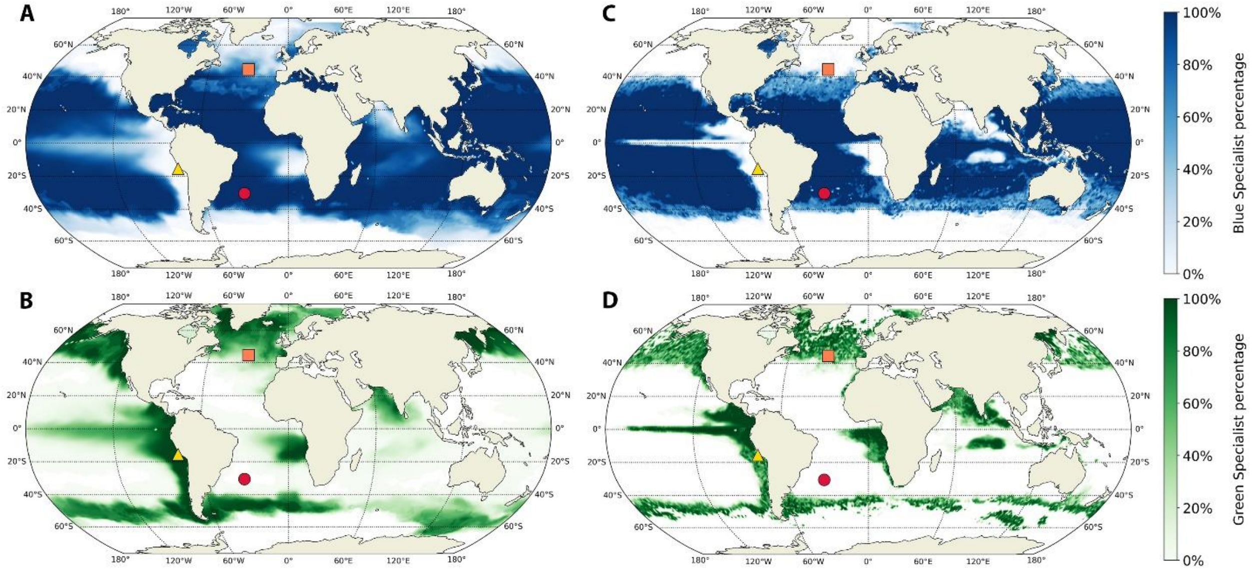

Fig. S5. Annual average biomass for *Synechococcus* pigment types as a percentage of total *Synechococcus* biomass for the specialist only simulations with (A,B) and without (C,D) advection. Disabling advection did not substantially alter pigment types percentage over the total *Synechococcus* biomass in the specialists-only simulation. In both the standard simulation and the no-advection simulation, BS and GS accounted for 75% and 25%, and 75% and 25% of the *Synechococcus* biomass, respectively. The three markers on the maps (yellow triangle, red circle and orange square) indicate the position of the representative upwelling, subtropical and temperate locations discussed in the main text.

The additional simulations encompassing different degrees of *Synechococcus* pigment diversity and the no-advection experiments underscored the added value of employing a complex 3D ecosystem model to investigate plankton distribution and competition. Specifically, these analyses elucidated the impact of evolutionary traits such as pigment diversity and chromatic acclimation within the context of the ocean physical environment on both distribution patterns and competitive dynamics.

Supplemental section 5: Model validation

In this study, the Matthews correlation coefficient was applied to assess the agreement between the dominant Synechococcus PT predicted by the Darwin model and those observed in the Tara Oceans metagenome dataset. The choice of this coefficient, combined with the emphasis on the dominant PT, enabled us to bridge the gap between the Darwin model output (carbon-based biomass) and the Tara Oceans dataset (relative abundance of Synechococcus pigment types as derived from the recruitment of three marker genes), which are not directly comparable. The Matthews correlation coefficient is a robust statistical metric designed to evaluate the agreement between predicted and observed categorical data (*39*). It is particularly advantageous in cases involving imbalanced datasets or multiple classes, outperforming simpler metrics like accuracy or Cohen's Kappa. Unlike accuracy, which can be misleading in skewed data distributions, or Cohen's Kappa, which does not fully account for the balance between all confusion matrix components, the Matthews correlation coefficient simultaneously considers true positives, false positives, true negatives, and false negatives (*58*). This ensures a comprehensive assessment of model

performance across all classes without bias toward the majority class. The Matthews correlation coefficient value ranges from −1 to +1, where +1 indicates perfect prediction, 0 reflects random chance, and −1 denotes complete disagreement between predictions and observations. This makes the Matthews correlation coefficient a reliable and interpretable metric, especially in complex or imbalanced scenarios (*59*, *60*).

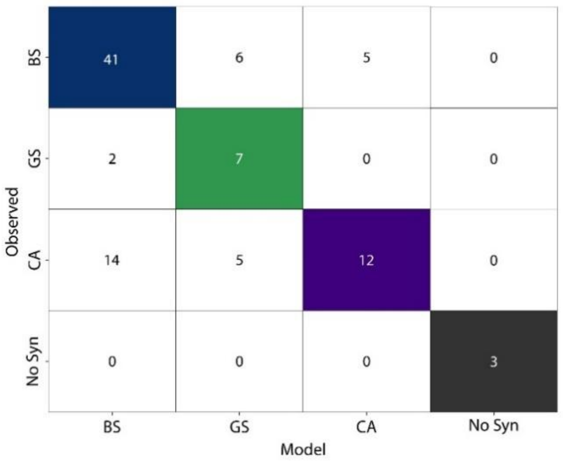

Fig. S6. Confusion matrix showing the agreement between the Darwin model and *Tara* Oceans stations in terms of dominant PT. The diagonal cells of the confusion matrix represent correct matches between the model simulation and in situ data for each category, whereas the off-diagonal cells indicate discrepancies. The Matthews correlation coefficient computed on the basis of this matrix is equal to 0.44.

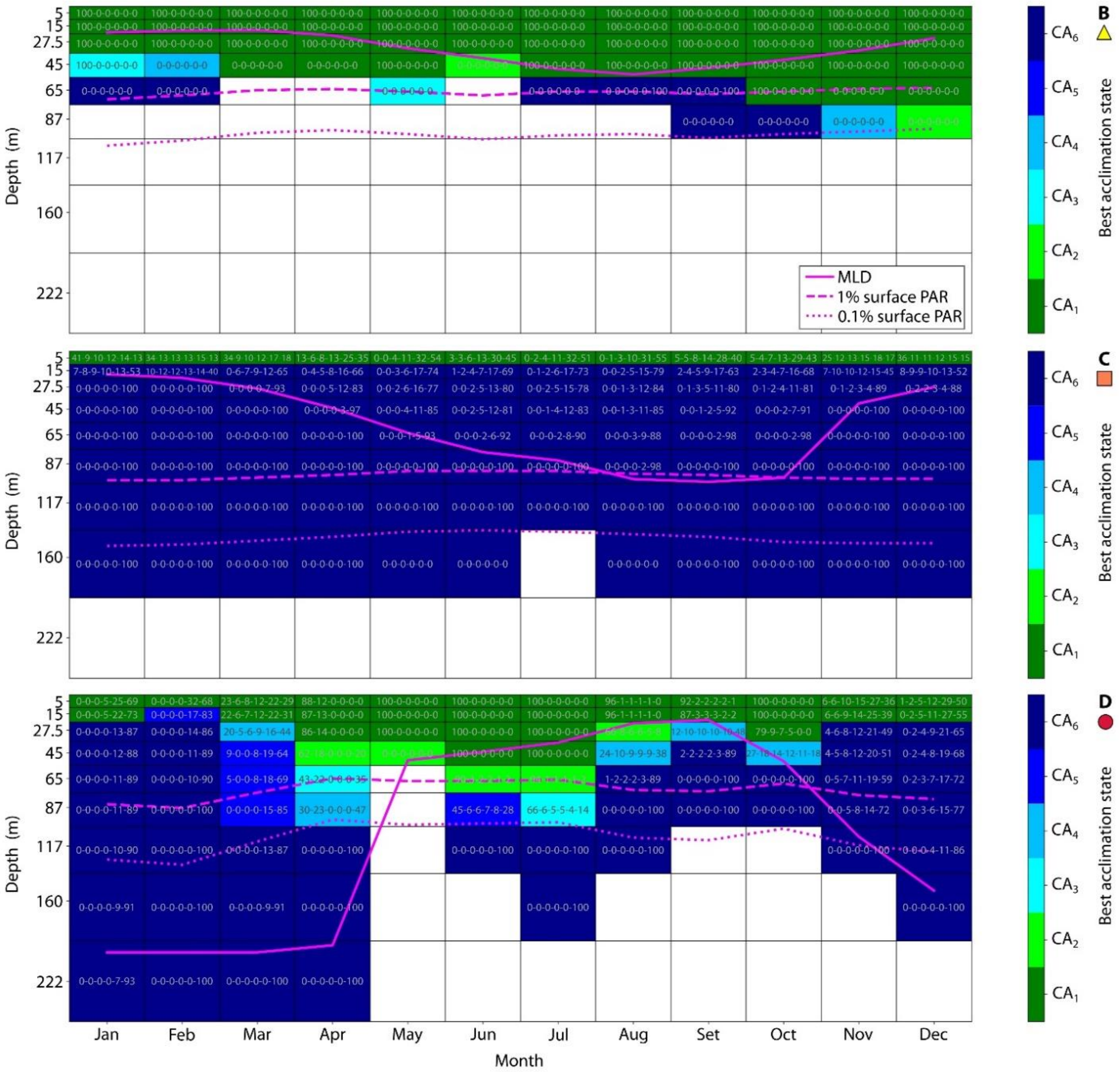

Fig. S7. Relative percentage of acclimation states. A,B,C, Monthly vertical profiles of the best acclimation state for the upwelling (**A**), subtropical (**B**) and temperate (**C**) regimes. The best acclimation state was the state most efficient in harvesting the available light for a given portion of the water column and month of the year, representing the acclimation target for all CAs in different states. The numbers within each depth box represent the relative contribution of each acclimation state to the total CA biomass, ordered from CA_1 (first number) to CA_6 (last number).

Table S1. Biomass differences between main simulation (BS + GS + CA) and supplemental simulations.

The table shows the differences in biomass between simulations with different numbers of *Synechococcus* pigment types. The additional simulations were compared to the main simulation (BS + GS + CA). Values are percentage difference, where positive numbers indicate higher values for the main simulation, and negative numbers indicate lower values. The three quantities evaluated were total, maximum and average *Synechococcus* integrated biomass over 200 m.

**Month**	**CA + BS + GS vs BS + GS**	**CA + BS + GS vs BS**	**CA + BS + GS vs GS**
**January**	-0.02%<br>+15.29%<br>+0.50%	-0.69%<br>+30.43%<br>-2.36%	-0.18%<br>+24.35%<br>+1.15%
**February**	-0.62%<br>+3.33%<br>+0.11%	-1.71%<br>-15.03%<br>-2.22%	+2.13%<br>-4.58%<br>+3.57%
**March**	+0.85%<br>+38.00%<br>+1.60%	+2.30%<br>+34.45%<br>+1.61%	+5.00%<br>+27.09%<br>+6.32%
**April**	+0.68%<br>+1.86%<br>+1.56%	+2.98%<br>+15.82%<br>+2.18%	+5.09%<br>+1.92%<br>+6.69%
**May**	+1.03%<br>+7.72%<br>+1.79%	+2.40%<br>+7.92%<br>+1.76%	+3.33%<br>+1.49%<br>+4.37%
**June**	+0.63%<br>+19.39%<br>+1.43%	+1.16%<br>+21.34%<br>+0.15%	+2.40%<br>+3.84%<br>+3.63%
**July**	+0.60%<br>+9.73%<br>+1.0%	+0.25%<br>+6.28%<br>-0.91%	+3.16%<br>+11.27%<br>+3.80%
**August**	-0.25%<br>-2.78%<br>+0.23%	+3.30%<br>+5.95%<br>+2.89%	+3.29%<br>+7.48%<br>+4.09%
**September**	+0.37%<br>-0.65%<br>+0.55%	+3.79%<br>+1.93%<br>+3.74%	+4.81%<br>+8.29%<br>+5.88%
**October**	+0.12%<br>+1.06%<br>+1.40%	+2.78%<br>-2.14%<br>+3.02%	+3.63%<br>+1.01%<br>+5.12%
**November**	+0.59%<br>+6.47%<br>+1.53%	+1.65%<br>+16.29%<br>+1.87%	+2.97%<br>+10.05%<br>+4.46%
**December**	+1.00%<br>+8.66%<br>+2.21%	+2.01%<br>+21.17%<br>+2.21%	+3.16%<br>+5.28%<br>+5.44%

Movie S1. Biogeography dynamics of simulated *Synechococcus*.

Top Row, Total *Synechococcus* biomass distribution integrated from surface to 200 m (first panel from the left) and dominant pigment type in terms of integrated biomass from surface to 200 m (second panel from the left). **Bottom row**, Percentage contribution relative to the total *Synechococcus* biomass integrated within the first 200 m for the blue specialist (first panel from the left), the green specialist (middle panel), and the chromatic acclimator (last panel from the left).

REFERENCES AND NOTES

1. M. Stomp, J. Huisman, F. de Jongh, A. J. Veraart, D. Gerla, M. Rijkeboer, B. W. Ibelings, U. I. A. Wollenzien, L. J. Stal, Adaptive divergence in pigment composition promotes phytoplankton biodiversity. *Nature* **432**, 104–107 (2004).

2. M. Stomp, J. Huisman, L. J. Stal, H. C. P. Matthijs, Colorful niches of phototrophic microorganisms shaped by vibrations of the water molecule. *ISME J.* **1**, 271–282 (2007).

3. C. S. Ting, G. Rocap, J. King, S. W. Chisholm, Cyanobacterial photosynthesis in the oceans: The origins and significance of divergent light-harvesting strategies. *Trends Microbiol.* **10**, 134–142 (2002).

4. V. M. Luimstra, J. M. H. Verspagen, T. Xu, J. M. Schuurmans, J. Huisman, Changes in water color shift competition between phytoplankton species with contrasting light-harvesting strategies. *Ecology* **101**, e02951 (2020).

5. A. Shukla, A. Biswas, N. Blot, F. Partensky, J. A. Karty, L. A. Hammad, L. Garczarek, A. Gutu, W. M. Schluchter, D. M. Kehoe, Phycoerythrin-specific bilin lyase–isomerase controls blue-green chromatic acclimation in marine *Synechococcus*. *Proc. Natl. Acad. Sci. U.S.A.* **109**, 20136–20141 (2012).

6. J. E. Sanfilippo, L. Garczarek, F. Partensky, D. M. Kehoe, Chromatic acclimation in cyanobacteria: A diverse and widespread process for optimizing photosynthesis. *Annu. Rev. Microbiol.* **73**, 407–433 (2019).

7. P. G. Falkowski, J. LaRoche, Acclimation to spectral irradiance in algae. *J. Phycol.* **27**, 8–14 (1991).

8. S. E. Voerman, B. C. Marsh, R. G. Bahia, G. H. Pereira-Filho, A. C. F. Becker, G. M. Amado-Filho, A. Ruseckas, G. A. Turnbull, I. D. W. Samuel, H. L. Burdett, Dominance of photo over chromatic acclimation strategies by habitat-forming mesophotic red algae. *Proc. R. Soc. B Biol. Sci.* **290**, 20231329 (2023).

9. M. Takahashi, K. Mikami, Blue–red chromatic acclimation in the red alga *Pyropia yezoensis*. *Algal Res.* **58**, 102428 (2021).

10. B. M. Wolf, R. E. Blankenship, Far-red light acclimation in diverse oxygenic photosynthetic organisms. *Photosynth. Res.* **142**, 349–359 (2019).

11. A. E. Hickman, S. Dutkiewicz, R. G. Williams, M. J. Follows, Modelling the effects of chromatic adaptation on phytoplankton community structure in the oligotrophic ocean. *Mar. Ecol. Prog. Ser.* **406**, 1–17 (2010).

12. A. E. Hickman, P. M. Holligan, C. M. Moore, J. Sharples, V. Krivtsov, M. R. Palmer, Distribution and chromatic adaptation of phytoplankton within a shelf sea thermocline. *Limnol. Oceanogr.* **54**, 525–536 (2009).

13. T. Grébert, H. Doré, F. Partensky, G. K. Farrant, E. S. Boss, M. Picheral, L. Guidi, S. Pesant, D. J. Scanlan, P. Wincker, S. G. Acinas, D. M. Kehoe, L. Garczarek, Light color acclimation is a key process in the global ocean distribution of *Synechococcus* cyanobacteria. *Proc. Natl. Acad. Sci.* **115**, E2010–E2019 (2018).

14. G. K. Farrant, H. Doré, F. M. Cornejo-Castillo, F. Partensky, M. Ratin, M. Ostrowski, F. D. Pitt, P. Wincker, D. J. Scanlan, D. Iudicone, S. G. Acinas, L. Garczarek, Delineating ecologically significant taxonomic units from global patterns of marine picocyanobacteria. *Proc. Natl. Acad. Sci. U.S.A.* **113**, E3365–E3374 (2016).

15. P. Flombaum, J. L. Gallegos, R. A. Gordillo, J. Rincón, L. L. Zabala, N. Jiao, D. M. Karl, W. K. W. Li, M. W. Lomas, D. Veneziano, C. S. Vera, J. A. Vrugt, A. C. Martiny, Present and future global distributions of the marine Cyanobacteria *Prochlorococcus* and *Synechococcus*. *Proc. Natl. Acad. Sci. U.S.A.* **110**, 9824–9829 (2013).

16. M. L. Paulsen, H. Doré, L. Garczarek, L. Seuthe, O. Müller, R.-A. Sandaa, G. Bratbak, A. Larsen, *Synechococcus* in the Atlantic gateway to the Arctic Ocean. *Front. Mar. Sci.* **3**, 191 (2016).

17. R. J. Olson, S. W. Chisholm, E. R. Zettler, E. V. Armbrust, Pigments, size, and distributions of *Synechococcus* in the North Atlantic and Pacific Oceans. *Limnol. Oceanogr.* **35**, 45–58 (1990).

18. A. M. Wood, D. A. Phinney, C. S. Yentsch, Water column transparency and the distribution of spectrally distinct forms of phycoerythrin-containing organisms. *Mar. Ecol. Prog. Ser.* **162**, 25–31 (1998).

19. W. A. Sidler, Phycobilisome and phycobiliprotein structures, in *The Molecular Biology of Cyanobacteria*, vol. 1 of *Advances in Photosynthesis*, D. A. Bryant, Ed. (Springer Netherlands, 1994), pp. 139–216; https://doi.org/10.1007/978-94-011-0227-8_7.

20. C. Six, J.-C. Thomas, L. Garczarek, M. Ostrowski, A. Dufresne, N. Blot, D. J. Scanlan, F. Partensky, Diversity and evolution of phycobilisomes in marine *Synechococcus spp.*: A comparative genomics study. *Genome Biol.* **8**, R259 (2007).

21. F. Humily, F. Partensky, C. Six, G. K. Farrant, M. Ratin, D. Marie, L. Garczarek, A gene island with two possible configurations is involved in chromatic acclimation in marine *Synechococcus*. *PLoS One* **8**, e84459 (2013).

22. T. Grébert, L. Garczarek, V. Daubin, F. Humily, D. Marie, M. Ratin, A. Devailly, G. K. Farrant, I. Mary, D. Mella-Flores, G. Tanguy, K. Labadie, P. Wincker, D. M. Kehoe, F. Partensky, Diversity and evolution of pigment types in marine *Synechococcus* cyanobacteria. *Genome Biol. Evol.* **14**, evac035 (2022).

23. L. J. Ong, A. N. Glazer, Phycoerythrins of marine unicellular cyanobacteria. I. Bilin types and locations and energy transfer pathways in *Synechococcus* spp. phycoerythrins. *J. Biol. Chem.* **266**, 9515–9527 (1991).

24. S. Sunagawa, L. P. Coelho, S. Chaffron, J. R. Kultima, K. Labadie, G. Salazar, B. Djahanschiri, G. Zeller, D. R. Mende, A. Alberti, F. M. Cornejo-Castillo, P. I. Costea, C. Cruaud, F. d'Ovidio, S. Engelen, I. Ferrera, J. M. Gasol, L. Guidi, F. Hildebrand, F. Kokoszka, C. Lepoivre, G. Lima-Mendez, J. Poulain, B. T. Poulos, M. Royo-Llonch, H. Sarmento, S. Vieira-Silva, C. Dimier, M. Picheral, S. Searson, S. Kandels-Lewis, Tara

Oceans Coordinators, C. Bowler, C. de Vargas, G. Gorsky, N. Grimsley, P. Hingamp, D. Iudicone, O. Jaillon, F. Not, H. Ogata, S. Pesant, S. Speich, L. Stemmann, M. B. Sullivan, J. Weissenbach, P. Wincker, E. Karsenti, J. Raes, S. G. Acinas, P. Bork, Structure and function of the global ocean microbiome. *Science* **348**, 1261359 (2015).

25. B. A. Ward, S. Collins, S. Dutkiewicz, S. Gibbs, P. Bown, A. Ridgwell, B. Sauterey, J. D. Wilson, A. Oschlies, Considering the role of adaptive evolution in models of the ocean and climate system. *J. Adv. Model. Earth Syst.* **11**, 3343–3361 (2019).

26. S. Dutkiewicz, P. W. Boyd, U. Riebesell, Exploring biogeochemical and ecological redundancy in phytoplankton communities in the global ocean. *Glob. Chang. Biol.* **27**, 1196–1213 (2021).

27. S. Dutkiewicz, A. E. Hickman, O. Jahn, S. Henson, C. Beaulieu, E. Monier, Ocean colour signature of climate change. *Nat. Commun.* **10**, 578 (2019).

28. M. J. Behrenfeld, R. T. O'Malley, E. S. Boss, T. K. Westberry, J. R. Graff, K. H. Halsey, A. J. Milligan, D. A. Siegel, M. B. Brown, Revaluating ocean warming impacts on global phytoplankton. *Nat. Clim. Chang.* **6**, 323–330 (2016).

29. S. Dutkiewicz, A. E. Hickman, O. Jahn, W. W. Gregg, C. B. Mouw, M. J. Follows, Capturing optically important constituents and properties in a marine biogeochemical and ecosystem model. *Biogeosciences* **12**, 4447–4481 (2015).

30. C. L. Follett, S. Dutkiewicz, F. Ribalet, E. Zakem, D. Caron, E. V. Armbrust, M. J. Follows, Trophic interactions with heterotrophic bacteria limit the range of *Prochlorococcus*. *Proc. Natl. Acad. Sci. U.S.A.* **119**, e2110993118 (2022).

31. S. Dutkiewicz, C. L. Follett, M. J. Follows, F. Henderikx-Freitas, F. Ribalet, M. R. Gradoville, S. N. Coesel, H. Farnelid, Z. V. Finkel, A. J. Irwin, O. Jahn, D. M. Karl, J. P. Mattern, A. E. White, J. P. Zehr, E. V. Armbrust, Multiple biotic interactions establish phytoplankton community structure across environmental gradients. *Limnol. Oceanogr.* **69**, 1086–1100 (2024).

32. A. Morel, Y.-W. Ahn, F. Partensky, D. Vaulot, H. Claustre, *Prochlorococcus* and *Synechococcus*: A comparative study of their size, pigmentation and related optical properties. *J. Mar. Res.* **51**, 617–649 (1993).

33. D. Stramski, A. Bricaud, A. Morel, Modeling the inherent optical properties of the ocean based on the detailed composition of the planktonic community. *Appl. Optics* **40**, 2929–2945 (2001).

34. E. Organelli, C. Nuccio, L. Lazzara, J. Uitz, A. Bricaud, L. Massi, On the discrimination of multiple phytoplankton groups from light absorption spectra of assemblages with mixed taxonomic composition and variable light conditions. *Appl. Optics* **56**, 3952–3968 (2017).

35. L. Dufour, L. Garczarek, B. Gouriou, J. Clairet, M. Ratin, F. Partensky, Differential acclimation kinetics of the two forms of type IV chromatic acclimaters occurring in marine *Synechococcus* cyanobacteria. *Front. Microbiol.* **15**, 1349322 (2024).

36. J. T. O. Kirk, *Light and Photosynthesis in Aquatic Ecosystems* (Cambridge Univ. Press, ed. 2, 1994); https://www.cambridge.org/core/books/light-and-photosynthesis-in-aquatic-ecosystems/C19B28AE07B1CDEBDA5593194DE4E304.

37. J. Neveux, F. Lantoine, D. Vaulot, D. Marie, J. Blanchot, Phycoerythrins in the southern tropical and equatorial Pacific Ocean: Evidence for new cyanobacterial types. *J. Geophys. Res. Oceans* **104**, 3311–3321 (1999).

38. N. D. Sherry, A. Michelle Wood, Phycoerythrin-containing picocyanobacteria in the Arabian Sea in February 1995:: Diel patterns, spatial variability, and growth rates. *Deep Sea Res. II Top. Stud. Oceanogr.* **48**, 1263–1283 (2001).

39. B. W. Matthews, Comparison of the predicted and observed secondary structure of T4 phage lysozyme. *Biochim. Biophys. Acta* **405**, 442–451 (1975).

40. K. R. Hunter-Cevera, A. F. Post, E. E. Peacock, H. M. Sosik, Diversity of *Synechococcus* at the Martha's vineyard coastal observatory: Insights from culture isolations, clone libraries, and flow cytometry. *Microb. Ecol.* **71**, 276–289 (2016).

41. X. Zhang, S. Cheung, J. Wang, G. Zhang, Y. Wei, H. Liu, J. Sun, H. Liu, Highly diverse *Synechococcus* pigment types in the Eastern Indian Ocean. *Front. Microbiol.* **13**, 806390 (2022).

42. T. Holtrop, J. Huisman, M. Stomp, L. Biersteker, J. Aerts, T. Grébert, F. Partensky, L. Garczarek, H. J. van der Woerd, Vibrational modes of water predict spectral niches for photosynthesis in lakes and oceans. *Nat. Ecol. Evol.* **5**, 55–66 (2021).

43. R. Lovindeer, L. J. Ustick, F. Primeau, A. C. Martiny, K. R. M. Mackey, Modeling ocean color niche selection by *Synechococcus* blue-green acclimaters. *J. Geophys. Res. Oceans* **126**, e2021JC017434 (2021).

44. W. W. L. Cheung, R. Watson, D. Pauly, Signature of ocean warming in global fisheries catch. *Nature* **497**, 365–368 (2013).

45. P. W. Boyd, S. T. Lennartz, D. M. Glover, S. C. Doney, Biological ramifications of climate-change-mediated oceanic multi-stressors. *Nat. Clim. Chang.* **5**, 71–79 (2015).

46. X. Xia, H. Liu, D. Choi, J. H. Noh, Variation of *Synechococcus* pigment genetic diversity along two turbidity gradients in the China seas. *Microb. Ecol.* **75**, 10–21 (2018).

47. B. B. Cael, K. Bisson, E. Boss, S. Dutkiewicz, S. Henson, Global climate-change trends detected in indicators of ocean ecology. *Nature* **619**, 551–554 (2023).

48. S. A. Henson, B. B. Cael, S. R. Allen, S. Dutkiewicz, Future phytoplankton diversity in a changing climate. *Nat. Commun.* **12**, 5372 (2021).

49. G. Kulk, T. Platt, J. Dingle, T. Jackson, B. F. Jönsson, H. A. Bouman, M. Babin, R. J. W. Brewin, M. Doblin, M. Estrada, F. G. Figueiras, K. Furuya, N. González-Benítez, H. G. Gudfinnsson, K. Gudmundsson, B. Huang, T. Isada, Ž. Kovač, V. A. Lutz, E. Marañón, M. Raman, K. Richardson, P. D. Rozema, W. H. van de Poll, V. Segura, G. H. Tilstone, J. Uitz,

V. van Dongen-Vogels, T. Yoshikawa, S. Sathyendranath, Primary production, an index of climate change in the ocean: Satellite-based estimates over two decades. *Remote Sens.* **12**, 826 (2020).

50. J. Marshall, A. Adcroft, C. Hill, L. Perelman, C. Heisey, A finite-volume, incompressible Navier Stokes model for studies of the ocean on parallel computers. *J. Geophys. Res. Oceans* **102**, 5753–5766 (1997).

51. S. Dutkiewicz, P. Cermeno, O. Jahn, M. J. Follows, A. E. Hickman, D. A. A. Taniguchi, B. A. Ward, Dimensions of marine phytoplankton diversity. *Biogeosciences* **17**, 609–634 (2020).

52. W. W. Gregg, N. W. Casey, Skill assessment of a spectral ocean–atmosphere radiative model. *J. Mar. Syst.* **76**, 49–63 (2009).

53. R. R. Bidigare, M. E. Ondrusek, J. H. Morrow, D. A. Kiefer, In-vivo absorption properties of algal pigments. *Proc. SPIE* **1302**, 290–302 (1990).

54. P. Stoica, P. Babu, Pearson–Matthews correlation coefficients for binary and multinary classification. *Signal Process.* **222**, 109511 (2024).

55. J. Ras, H. Claustre, J. Uitz, Spatial variability of phytoplankton pigment distributions in the Subtropical South Pacific Ocean: Comparison between in situ and predicted data. *Biogeosciences* **5**, 353–369 (2008).

56. D. Stramski, R. A. Reynolds, S. Kaczmarek, J. Uitz, G. Zheng, Correction of pathlength amplification in the filter-pad technique for measurements of particulate absorption coefficient in the visible spectral region. *Appl. Optics* **54**, 6763–6782 (2015).

57. E. T. Buitenhuis, M. Vogt, R. Moriarty, N. Bednaršek, S. C. Doney, K. Leblanc, C. Le Quéré, Y.-W. Luo, C. O'Brien, T. O'Brien, J. Peloquin, R. Schiebel, C. Swan, MAREDAT: Towards a world atlas of MARine Ecosystem DATa. *Earth Syst. Sci. Data* **5**, 227–239 (2013).

58. D. Chicco, M. J. Warrens, G. Jurman, The Matthews correlation coefficient (MCC) is more informative than Cohen's Kappa and brier score in binary classification assessment. *IEEE Access* **9**, 78368–78381 (2021).

59. D. Chicco, G. Jurman, The advantages of the Matthews correlation coefficient (MCC) over F1 score and accuracy in binary classification evaluation. *BMC Genomics* **21**, 6 (2020).

60. G. Jurman, S. Riccadonna, C. Furlanello, A comparison of MCC and CEN error measures in multi-class prediction. *PLOS ONE* **7**, e41882 (2012).
